# Silver Nanoparticles Impair Cognitive Functions and Modify the Hippocampal Level of Neurotransmitters in a Coating-Dependent Manner

**DOI:** 10.3390/ijms222312706

**Published:** 2021-11-24

**Authors:** Katarzyna Dziendzikowska, Małgorzata Węsierska, Joanna Gromadzka-Ostrowska, Jacek Wilczak, Michał Oczkowski, Sylwia Męczyńska-Wielgosz, Marcin Kruszewski

**Affiliations:** 1 Chair of Nutrition Physiology, Department of Dietetics, Institute of Human Nutrition Sciences, Warsaw University of Life Sciences—SGGW, Nowoursynowska 159C, 02-776 Warsaw, Poland; joanna_gromadzka_ostrowska@sggw.edu.pl (J.G.-O.); michal_oczkowski@sggw.edu.pl (M.O.); 2 Laboratory of Neurophysiology, Nencki Institute of Experimental Biology, Polish Academy of Sciences, 3 Pasteur Street, 02-093 Warsaw, Poland; 3 Department of Physiological Sciences, Institute of Veterinary Medicine, Warsaw University of Life Sciences, Nowoursynowska 159, 02-776 Warsaw, Poland; jacek_wilczak@sggw.edu.pl; 4 Centre for Radiobiology and Biological Dosimetry, Institute of Nuclear Chemistry and Technology, Dorodna 16, 03-195 Warsaw, Poland; s.meczynska@ichtj.waw.pl (S.M.-W.); m.kruszewski@ichtj.waw.pl (M.K.); 5 Department of Molecular Biology and Translational Research, Institute of Rural Health, Jaczewskiego 2, 20-090 Lublin, Poland

**Keywords:** silver nanoparticles, nanoparticle coating, active allothetic place avoidance task, spatial long- and short-term memory, hippocampus, neurotransmitters, inflammation

## Abstract

Due to their potent antibacterial properties, silver nanoparticles (AgNPs) are widely used in industry and medicine. However, they can cross the brain–blood barrier, posing a risk to the brain and its functions. In our previous study, we demonstrated that oral administration of bovine serum albumin (BSA)-coated AgNPs caused an impairment in spatial memory in a dose-independent manner. In this study, we evaluated the effects of AgNPs coating material on cognition, spatial memory functioning, and neurotransmitter levels in rat hippocampus. AgNPs coated with BSA (AgNPs(BSA)), polyethylene glycol (AgNPs(PEG)), or citrate (AgNPs(Cit)) or silver ions (Ag^+^) were orally administered at a dose of 0.5 mg/kg b.w. to male Wistar rats for a period of 28 days, while the control (Ctrl) rats received 0.2 mL of water. The acquisition and maintenance of spatial memory related to place avoidance were assessed using the active allothetic place avoidance task, in which rats from AgNPs(BSA), AgNPs(PEG), and Ag^+^ groups performed worse than the Ctrl rats. In the retrieval test assessing long-term memory, only rats from AgNPs(Cit) and Ctrl groups showed memory maintenance. The analysis of neurotransmitter levels indicated that the ratio between serotonin and dopamine concentration was disturbed in the AgNPs(BSA) rats. Furthermore, treatment with AgNPs or Ag^+^ resulted in the induction of peripheral inflammation, which was reflected by the alterations in the levels of serum inflammatory mediators. In conclusion, depending on the coating material used for their stabilization, AgNPs induced changes in memory functioning and concentration of neurotransmitters.

## 1. Introduction

Due to their antibacterial properties, silver nanoparticles (AgNPs) are extensively used for in the production of goods, such as health care products and food-related materials, including edible films, food packaging, and containers [1]. The unique biomedical properties of AgNPs have also enabled their wide application in medical products, and as a component of, for example, wound and burn healing gels, drug carriers, and implants and prostheses [2]. The utilization of AgNPs in the preparation of various consumer and medical products has raised concerns about their potential adverse effects on human health.

The increasing use of nanomaterials, including AgNPs, puts the environment at risk, which may lead to negative consequences on organisms, including humans. In vitro experiments have indicated that AgNPs penetrated the cells of living organisms and accumulated in large amounts within the endosomes, lysosomes, cytoplasm, and mitochondria [3]. In addition, several in vitro and in vivo studies on animal models demonstrated that exposure to AgNPs may be associated with genotoxic and cytotoxic effects on the nervous system [4,5]. It was also reported that nanoparticles exhibited the ability to pass the blood–brain barrier (BBB) and translocate to the brain [6], affecting the functions of brain cells and disrupting the homeostasis of the central nervous system (CNS). Fuster et al. indicated that exposure to AgNPs at noncytotoxic concentrations caused changes in several molecular pathways related to inflammation, cellular repair, regeneration, and induced an oxidative stress response [7]. Our previous research showed that AgNPs administered to the body were able to enter and accumulate in different tissues and organs, including the brain [8]. Although the brain is not considered responsible for Ag retention in the body, the results of our research indicated that AgNPs redistributed from the initial retention organs, such as the liver, spleen, and lungs, and translocated and accumulated in the brain [8]. Interestingly, after oral exposure, the concentration of silver was significantly higher in the hippocampus compared to the lateral and frontal cortex or cerebellum. Surprisingly, our study revealed that silver was found in an ionic form rather than as nanoparticles, suggesting the crucial role of silver ions (Ag^+^) in AgNPs-induced impairment of higher brain functions, which was confirmed by the observed detrimental effects on memory and cognitive coordination processes [9].

Thus far, the effects of AgNPs accumulation on brain functions, such as cognitive performance, learning, and memory, and on the mechanisms and factors affecting susceptibility to nanoparticles-induced malfunction of CNS are not completely understood. Recent studies indicated that AgNPs deteriorated learning and social activity in mice and disturbed the functions of the hippocampus in rats [4,10]. Furthermore, AgNPs were also shown to potentially alter the functions of neuronal cells and induce synaptic degeneration, while inhibiting the expression of genes associated with neurotransmitter metabolism, oxidative stress, and inflammation [11,12,13,14]. However, it is still unclear whether AgNPs-induced dysfunction of memory and cognitive coordination is related to the coating material of the nanoparticles and/or the ability of the nanoparticles to release Ag^+^ after oral administration. Studies showed that the toxic effect of metal nanoparticles is determined by the route and duration of exposure, particle size, dose, and surface coating. Therefore, in this study, we aimed to investigate the effect of AgNPs coating on the acquisition and maintenance of spatial memory tested in the active allothetic place avoidance task (AAPAT) and neurotransmitter levels in rat hippocampus. Additionally, we assessed the effects of particulate and ionic forms of silver on systemic toxicity and inflammatory marker levels.

## 2. Results

### 2.1. General Health Status

**Figure 1:**
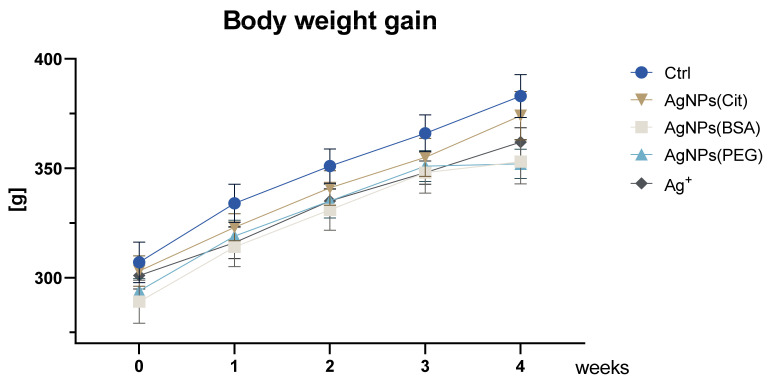
Body weight gain of rats during the experiment (mean ± SEM). (two-way repeated measures ANOVA: effect of group, *F*_4,33_ = 1.56, NS; effect of day, *F*_4,132_ = 446.05, *p* = 0.000; effect of group-by-day interaction, *F*_4,132_ = 2.11, *p* = 0.011).

In the present study, we examined neurotoxicity of AgNPs with different coating and Ag^+^ ions on rats. The study was designed in accordance with OECD 407 guidelines [15] regarding Toxicity Studies in Rodents. Body weight gain (weekly) and general clinical observations (daily) were recorded for the whole duration of the experiment. The general health status of animals was evaluated in order to verify systemic effects of exposure to relatively low doses of AgNPs or Ag^+^ ions. During the experiment, no adverse effects of toxicity were observed. All animals showed steady development and growth in all experimental groups. During the experiment, the body weight of the rats in all the studied groups systematically increased (ANOVA, time *p* < 0.001) (Figure 1). Statistical analysis revealed that the weight gain of rats was significantly dependent on the interaction between the analyzed factors (time vs. treated group; ANOVA, *p* = 0.011). The Tukey post hoc test showed that the weight was significantly increased from week 1 to week 3 of the experiment in all the groups (*p* < 0.05, for all comparisons). However, no increase in body mass was found between weeks 2 and 3 in the rats from the Ag^+^ group. In week 4, a significant increase in body weight was noted in the Ctrl, AgNPs(Cit), and Ag^+^ group (*p* < 0.01, for all comparisons). On the other hand, the body weight of rats in the AgNPs(BSA) and AgNPs(PEG) groups did not differ significantly from the weight noted in week 3 of the experiment, which suggests that weight gain was inhibited in these animals.

#### 2.1.1. Effect of Nanoparticle Coating on Hematological Parameters

At the end of the experiment, relevant hematological parameters were analyzed in order to evaluate the general health status of rats. None of the examined parameters exceeded the reference values [16]. However, the statistical analysis of the RBC parameters confirmed that AgNPs coating had an influence on HGB (ANOVA, *p* = 0.016), MCH (ANOVA, *p* = 0.003) and MCHC (ANOVA, *p* = 0.003), MCV (ANOVA, *p* = 0.004), and RDWc (ANOVA, *p* = 0.003) (Table 1). The Tukey post hoc test confirmed that the MCV of the Ag^+^ group was the smallest in comparison to the Ctrl group (*p* < 0.05) and MCH was the smallest in comparison to the AgNPs(BSA), AgNPs(PEG), and AgNPs(Cit) group (*p* < 0.01, *p* < 0.01, and *p* < 0.05, respectively), whereas the RDWc of the Ag^+^ group was the highest (*p* < 0.01 for Ag^+^ vs. AgNPs(BSA) group and *p* < 0.001 Ag^+^ vs. AgNPs(PEG) group). Additionally, rats receiving Ag^+^ showed the lowest concentration of blood HGB in comparison to the animals receiving AgNPs(BSA) (*p* < 0.05). The RBC and HCT values did not differ between the studied groups (ANOVA, NS). Similarly, the values of RBC parameters did not differ significantly between the groups receiving nanosilver with different types of coating and the Ctrl group.

The platelet number and other platelet indices, including plateletcrit (PCT) and platelet volume distribution width, also did not significantly differ between the silver-treated groups and the Ctrl group. The total number of WBC significantly varied between the groups depending on the type of nanosilver coating (ANOVA, *p* = 0.042), with the highest value observed in the AgNPs(Cit) group. Only the rats in this group had a higher WBC value compared to the Ctrl group (*p* < 0.05). The values of platelet and WBC parameters are listed in Table 1.

#### 2.1.2. Effect of Nanoparticle Coating on Blood Plasma Cytokine Concentration

The Rat Cytokine 24-Plex Panel assay was used to assess whether AgNPs or Ag^+^ induce systemic inflammation. The results showed that the blood plasma concentration of most of the investigated cytokines was changed after the 28-day administration of AgNPs with different types of coating or Ag^+^ (Table 2). The ANOVA results were confirmed by post hoc test, which revealed that the plasma concentrations of IL-5 (*p* < 0.01), IL-10 (*p* < 0.01), IL-12(p70) (*p* < 0.001), M-CSF (*p* < 0.001), and TNF-α (*p* < 0.01) were significantly higher in the AgNPs(PEG) group in comparison to the Ctrl group. The level of IFN-γ, a protein involved in inducing TNF-α secretion and stimulating the release of reactive oxygen species from macrophages, was found to be elevated in the AgNPs(PEG) and AgNPs(Cit) groups vs. the Ctrl group (*p* < 0.01 and *p* < 0.05, respectively). The level of pro-inflammatory IL-6 was also significantly increased in the AgNPs(PEG) and AgNPs(BSA) groups in comparison to the Ctrl group (*p* < 0.01 and *p* < 0.05, respectively). The level of IL-12 protein was significantly increased in the rats receiving AgNPs(PEG), AgNPs(Cit), and Ag^+^ compared to the Ctrl rats (*p* < 0.001, *p* < 0.001, and *p* < 0.01, respectively). The rats in the AgNPs(PEG) group showed significantly higher concentration of IL-1β (vs AgNPs(BSA) group, *p* < 0.001) and TNF-α (vs Ag^+^ group, *p* < 0.001), as well as some chemotactic factors of immune cells including GM-CSF (vs AgNPs(BSA) group, *p* < 0.01), G-CSF (vs Ag^+^ group, *p* < 0.05), and M-CSF (vs AgNPs(BSA) and Ag^+^ groups, *p* < 0.001 and *p* < 0.05, respectively). An increased concentration of other proinflammatory cytokines (IL-1α, IL-2, and IL-4) was observed in the rats from the AgNPs(PEG) group, but the changes were statistically insignificant. The concentrations of IL-7, IL-13, IL-18, MIP-1α, GRO/KC, and RANTES did not differ between the studied groups (Table 2).

Moreover, the AgNPs(BSA) group exhibited a lower concentration of IL-1β (vs AgNPs(PEG) group, *p* < 0.001) and M-CSF (vs AgNPs(PEG) group, *p* < 0.001). The Ag^+^ group had a higher plasma concentration of MIP-3α (vs Ctrl, AgNPs(Cit), and AgNPs(BSA) groups, *p* < 0.05, *p* < 0.01, and *p* < 0.05, respectively) and a lower concentration of G-CSF (vs AgNPs(PEG) group, *p* < 0.05) (Table 2). The concentrations of IL-17A, erythropoietin, and VEGF were found to be under detection limit.

### 2.2. Behavioral Data

#### 2.2.1. Acquisition of Spatial Memory

**Figure 2:**
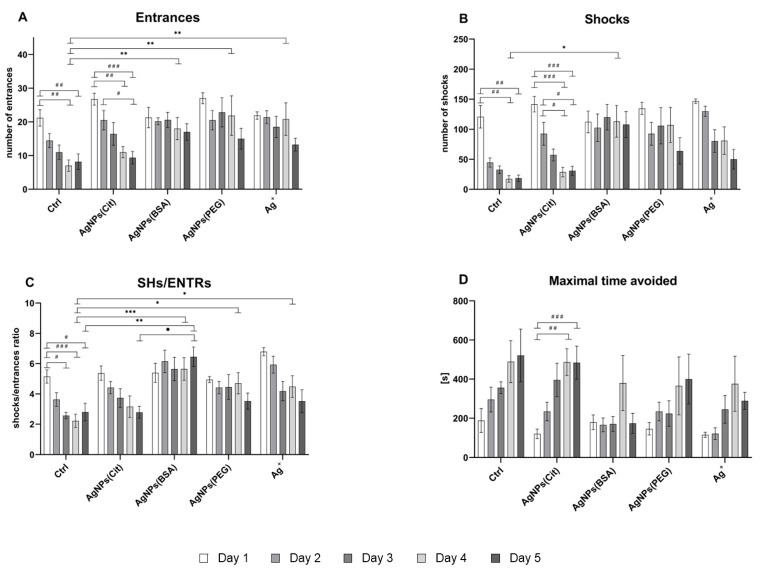
Behavioral measures of spatial memory assessed on 5 days of the active allothetic place avoidance training in rats exposed to AgNPs with different types of coating (citrate, BSA, and PEG) or Ag^+^; Ctrl rats received water. Memory acquisition: (**A**)—number of entrances (two-way repeated measures ANOVA: effect of group, *F*_4,22_=4.18, *p* = 0.006; effect of day, *F*_4,88_ = 15.97, *p* = 0.000; effect of group-by-day interaction, *F*_4,88_ = 2.11, *p* = 0.01); (**B**)—number of shocks (the same ANOVA: effect of group, *F*_4,25_ = 3.61, *p* = 0.019; effect of day, *F*_4,100_ = 20.17, *p* = 0.001; effect of group-by-day interaction, *F*_4,100_ = 2.08, *p* = 0.015); skill learning: (**C**)—SHs/ENTR ratio (the same ANOVA: effect of group, *F*_4,29_ = 10.21, *p* = 0.001; effect of day, *F*_4,116_ = 9.83, *p* = 0.001); short-term memory: (**D**)—Tmax [s] (the same ANOVA: effect of group, *F*_4,29_ = 3.27, *p* = 0.025; effect of day, *F*_4,116_ = 12.40, *p* = 0.001) (mean ± SEM). *, **, and ***: Significantly different from the Ctrl group at the same time point (* *p* < 0.05, ** *p* <0.001, and *** *p* < 0.001) (Tukey post hoc test). ^#^, ^##^, and ^###^: Significantly different within the same experimental group at different time points (^#^ *p* < 0.05, ^##^ *p* < 0.001, and ^###^ *p* < 0.001) (Tukey post hoc test). ^●^ Significantly different between AgNPs(Cit) group and AgNPs(BSA) group on 5 days of memory acquisition (^●^ *p* < 0.05) (Tukey post hoc test).

After 28 days of Ag administration, spatial memory acquisition was measured as the number of entrances into the shock sector and number of shocks on the consecutive 5 days of the place avoidance training. A decrease in both these values indicates memory acquisition. It was observed that the results strongly varied between the groups, which indicates the influence of the coatings used for AgNPs stabilization (Figure 2A,B).

However, the number of entrances into the shock sector was found to be significantly decreased after the third day of training in all groups (Tukey post hoc test for days, *p* < 0.001). Treatment with AgNPs(BSA), AgNPs(PEG), and Ag^+^ resulted in a higher number of entrances in comparison to Ctrl (Figure 2A). Rats from AgNPs(Cit) and Ctrl groups less frequently entered the to-be-avoided sector on day 4 and day 5 compared to day 1 (Tukey post hoc test for group-by-day interaction, *p* < 0.01). No differences were found in the number of entrances between the Ctrl and AgNPs(Cit) groups on day 4 and day 5, and the number significantly decreased compared to that measured on the initial days of training. On the other hand, the number of entrances was high and similar in AgNPs(BSA), AgNPs(PEG), and Ag^+^ groups on all days of acquisition training.

The number of shocks received on the to-be-avoided place was also found to be dependent on the treatment and varied on different days of training. Rats from the AgNPs(BSA), AgNPs(PEG), and Ag^+^ groups received more shocks than the Ctrl and AgNPs(Cit) rats (Figure 2B). On day 4 and day 5, the number of received shocks was lower compared to that on day 1–3 (Tukey post hoc test for days, *p* < 0.001). On day 4, AgNPs(BSA), AgNPs(PEG), and Ag^+^ rats received a higher number of shocks. Rats in the Ctrl group received a lower number of shocks than those in the AgNPs(BSA), Ag^+^, and AgNPs(PEG) groups on day 3 (post hoc test for group-by-day interaction, *p* < 0.01).

Proper performance in the place avoidance task requires skill learning ability, which is measured as the SHs/ENTR ratio (Figure 2C). A low SHs/ENTR ratio indicated effective skill learning. Among the studied groups, the ratio was significantly higher in the AgNPs(BSA) rats compared to the Ctrl rats, whereas rats from the AgNPs(Cit) group presented a similar ratio as the Ctrl rats, which was lower than that of the Ag^+^ or AgNPs-treated groups (Figure 2C). The rats from control group learned rapidly within the first session and maintained the progress over the long-time between next sessions. Contrarily, rats from AgNPs(BSA) group revealed impairment of skill learning

The values of Tmax measured on each day of the place avoidance training corresponded to the short-term memory (Figure 2D). Rats from all the treated groups presented worse Tmax than the Ctrl rats. The duration of Tmax was found to be significantly increased on the last two days of training compared to the first three days (Tukey post hoc test for days, *p* < 0.03).

#### 2.2.2. Long-Term Memory Maintenance

**Figure 3:**
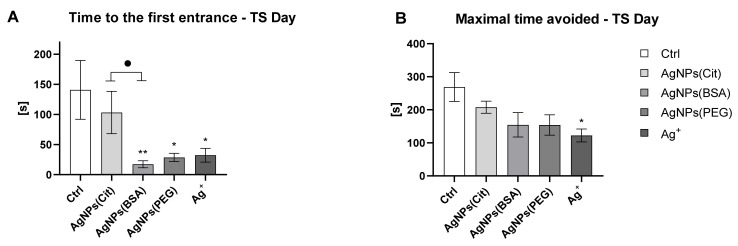
Retrieval test on long-term memory. (**A**)—T1 measured on test (TS) day (one-way ANOVA: effect of group, *F*_4,30_ = 5.91, *p* = 0.001) (mean ± SEM); (**B**)—Tmax measured on TS day (one-way ANOVA: effect of group, *F*_4,30_ = 5.91, *p* = 0.001) (mean ± SEM). * and **: Significantly different from the Ctrl group (* *p* < 0.05 and ** *p* < 0.01) (Tukey post hoc test). ^●^ Significantly different between the AgNPs(Cit) and AgNPs(BSA) group (^●^ *p* < 0.05) (Tukey post hoc test).

The strength of long-term memory maintenance was assessed over four days after the last day of the place avoidance training. A retrieval test was conducted without involving any shock presentation, and T1 was estimated. It was observed that rats from the AgNPs(BSA), AgNPs(PEG), and Ag^+^ groups made first entrances with a shorter T1 than Ctrl rats, whereas AgNPs(Cit)-treated rats presented a similar T1 as that of the Ctrl rats (Figure 3A). AgNPs(Cit) group presented a longer T1 in comparison to the AgNPs(BSA) group (*p* < 0.02). Importantly, the T1 of AgNPs(BSA), AgNPs(PEG), and Ag^+^ rats was in the range of 17.5–28.5 s, while that of Ctrl and AgNPs(Cit) was approximately 120 s. As the arena rotated one revolution per 60 s, a lack of memory retrieval was noted in rats from AgNPs(BSA), AgNPs(PEG), and Ag^+^ groups. This indicated that the rats from AgNPs(BSA), AgNPs(PEG), and Ag^+^ groups presented a lack of memory retrieval. In addition, Ag^+^ rats presented a significantly shorter Tmax than Ctrl rats (Figure 3B).

### 2.3. Effect of Nanoparticle Coating on Hippocampal Neurotransmitter Concentration

**Figure 4:**
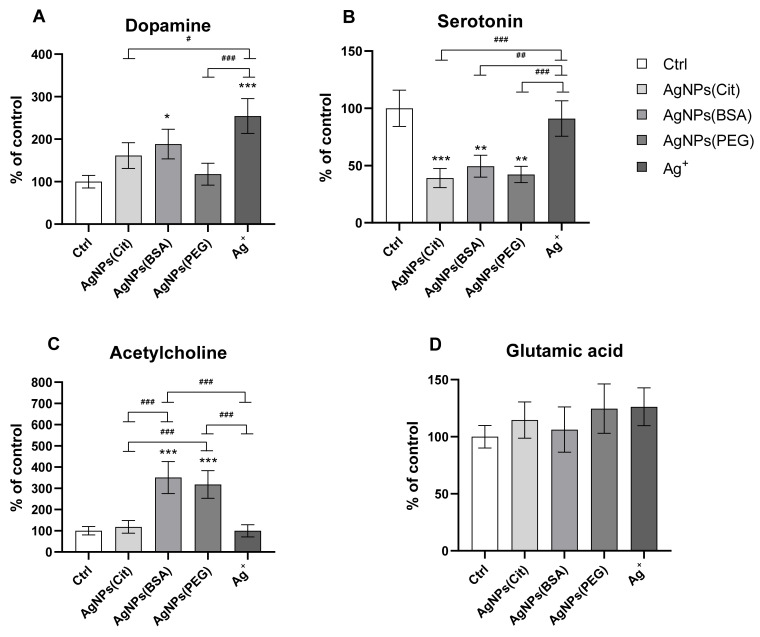
Levels of neurotransmitters in the hippocampus of rats exposed to AgNPs with different types of coating (citrate, BSA, and PEG) or Ag^+^. (**A**)—dopamine (one-way ANOVA: *F*_4,23_ = 10.98, *p* = 0.001); (**B**)—serotonin (one-way ANOVA: *F*_4,23_ = 15.06, *p* = 0.001); (**C**)—acetylcholine (one-way ANOVA: *F*_4,24_ = 28.37, *p* = 0.001); (**D**)—glutamic acid (one-way ANOVA: *F*_4,24_ = 0.67, NS) (% of Ctrl ± SEM). *, **, and ***: Significantly different from the Ctrl group (* *p* < 0.05, ** *p* < 0.001, and *** *p* < 0.001) (Tukey post hoc test). ^#^, ^##^, and ^###^: Significant differences between the groups exposed to AgNPs or Ag^+^ (^#^ *p* < 0.05, ^##^ *p* < 0.001, and ^###^ *p* < 0.001) (Tukey post hoc test).

The statistical analysis revealed that the hippocampal concentration of dopamine, serotonin, and acetylcholine was highly dependent on the type of AgNPs coating (ANOVA, *p* = 0.001; Figure 4), whereas the concentration of glutamic acid did not differ between the studied groups.

In the Ag^+^ and AgNPs(BSA) groups, the concentration of dopamine was found to be higher and similar to that of the Ctrl group (*p* < 0.001 and *p* < 0.5, respectively). By contrast, the concentration of this monoamine was generally low in the AgNPs(Cit)- and AgNPs(PEG)-treated groups, and much lower compared to the Ag^+^ group (*p* < 0.001 and *p* < 0.5, respectively) (Figure 4A).

The hippocampal serotonin concentration was also dependent on the AgNPs used for treatment, with the highest concentration observed in the Ctrl group. The serotonin concentration was lower in the AgNPs(BSA), AgNPs(PEG), and AgNPs(Cit) groups in comparison to the Ctrl group (*p* < 0.01, *p* < 0.01, and *p* < 0.001, respectively) (Figure 4B). Rats from AgNPs(BSA) and AgNPs(PEG) groups showed a higher acetylcholine concentration in the hippocampus than the rats from Ctrl, AgNPs(Cit), and Ag^+^ groups (*p* < 0.001, for all comparisons), while no differences in concentration were observed between AgNPs(Cit) as well as Ag^+^ and Ctrl groups (Figure 4C). The hippocampal concentration of glutamic acid was similar in all the analyzed groups (Figure 4D).

### 2.4. Fisher’s Linear Discriminant Analysis

**Figure 5:**
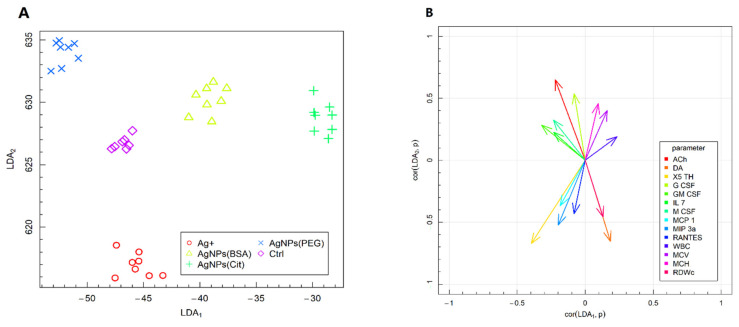
Fisher’s LDA: (**A**)—experimental data on the plane spanned by two of the most data-separating LDAs; (**B**)—parameters contributing the most to LDAs.

The results of Fisher’s LDA of the experimental data are illustrated in Figure 5. The analysis was performed to summarize the findings and obtain linear combinations (Linear Discriminants) of the analyzed parameters that allow the best distinction of the experimental groups included in the in vivo study model. The vectors presented in Figure 5B highlight the correlations between the relevant parameter values and two of the most data-separating combinations (LDA_1_ and LDA_2_). In addition, they indicate the direction in which corresponding parameters determine the separation of experimental groups presented in Figure 5A. The conducted LDA also supported the variance analysis.

Fisher’s LDA revealed that the factors that most correlated with combinations LDA_1_ and LDA_2_ differentiating the experimental groups were: (1) the levels of acetylcholine, serotonin, and dopamine among the neurotransmitters in the hippocampus; (2) the levels of G-CSF, GM-CSF, IL-7, M-CSF, MCP-1, MIP-3α, and RANTES among the plasma cytokines; and (3) the values of MCV, MCH, and RDWc among the hematological parameters (Figure 5B). The Ctrl group was separated from the other groups, regardless of the form of silver administered (Figure 5A). This differentiation is due to the value of the LDA_1_ coefficient, which is the most correlated with the following parameters: WBC, dopamine (DA), RDWc, and MCV (positively correlated with LDA_1_), as well as with serotonin (X5 TH) and M-CSF (negatively correlated with LDA_1_). The Ag^+^ group was separated based on the value of the LDA_2_ coefficient, which is the most correlated with acetylcholine and G-CSF (positively correlated with LDA_2_) as well as with X5 TH, DA, and MIP-3α (negatively correlated with LDA_1_). Importantly, the Ag^+^ group was visibly separated from the other groups receiving nanoparticles, which is reflected by its position relative to the vertical axis, showing the LDA_2_ coefficient, in Figure 5A. This position of the Ag^+^ group is mostly related to the values of RBC parameters, including MCHC, HGB, and HCT.

## 3. Discussion

Over the last decade, AgNPs have become one of the most popular nanomaterials and are found in about 30% of nanoparticle-containing products. However, a large number of scientific evidences have indicated that AgNPs can be toxic to different organs in the body, including the CNS, although these nanoparticles have antimicrobial, antiviral, and antifungal effects [17,18,19]. Thus, in this study, we investigated the effect of administration of AgNPs with different coatings at environmentally relevant concentrations on higher brain functions. Based on the available literature, we selected the oral route for administering AgNPs as it is one of the most critical routes of environmental nanoparticle exposure [20]. Despite difficulties in predicting the actual level of human exposure, it has been estimated that the exposure level can be 1.3–2.7 μg/kg b.w./day for adults and 1.6–3.5 μg/kg b.w./day in the case of children [21]. In the current experiment we decided to use a very low (0.5 mg/kg bw) dose of AgNPs. In the previous studies, we used a dose of AgNPs consistent with NOAEL value for oral exposure of rats (30 mg/kg b.w.) a significantly lower dose of 1 mg/kg b.w. As our findings indicated that oral exposure to the low dose of AgNPs induced memory impairment [9], in the present study, we further lowered the administered dose. NOAEL of 30 mg/kg corresponds to the human equivalent dose (HED) of 4.839 mg/kg for oral exposure [22]. The dose 0.5 mg/kg b.w. used in the present study corresponds to the HED of 0.081 mg/kg and is at a similar level to the predicted actual level of human exposure.

It is clear from the literature data that the toxicity of nanoparticles is determined by various factors, of which the type of applied coating is considered important. However, the exact influence of coatings on the toxicity of nanoparticles has not been fully investigated to date. In the preparation of nanoparticles for biomedical applications, various substances, such as citrate, PEG, polyvinylpyrrolidone (PVP), and proteins, are used as coating to improve the stability of nanoparticles, reduce their interaction with blood proteins, and ensure prolonged blood circulation [23]. Thus, in the present study, spherical AgNPs were coated with three different nanosilver surface coatings, namely BSA, citrate, or PEG. Moreover, AgNO_3_ was used as a source of Ag^+^ for comparison. Spherical AgNPs coated with the investigated coating materials have been widely used for several applications [13,23,24,25].

In recent years, AgNPs with different types of coating have been analyzed to determine their neurotoxicity [4,18]. However, the neurobehavioral effects of different coatings have not been clearly assessed under comparable conditions. Therefore, for the first time, we investigated the effect of AgNPs coated with different materials on higher brain functions. Our study demonstrated that the effect of nanosilver on cognitive functions was dependent on the coating material. Application of BSA-coated AgNPs resulted in the attenuation of cognitive functions, which in turn affected the short- and long-term memory. Application of PEG-coated AgNPs mainly influenced short-term memory, while citrate-coated AgNPs or water exhibited the opposite effect. Additionally, administration of Ag^+^ led to ineffective learning, causing debilitation of short- and long-term memory. On the other hand, the experimental path length data showed no effect on locomotion. The effect of AgNPs on long-term allothetic spatial memory was studied earlier based on the Morris water maze task. In this task, contrary to the AAPA all available information was used by animals for memory acquisition. The observed results indicated that exposure to uncoated AgNPs allowed memory maintenance at almost a similar level as the control in mice [26], but memory was found to be impaired in the case of rats [27]. This discrepancy can be related to differences in the routes of AgNPs administration (intraperitoneal vs. nasal), dose of AgNPs administered (10, 25, and 50 mg/kg b.w. vs. 3 or 30 mg/kg b.w.), and rodent species tested (mice vs. rats). Furthermore, it was demonstrated in mice that intranasal application of AgNPs in water suspension had no effect on the long-term allothetic memory which was determined by the distance to the platform in the Morris water maze test, although AgNPs-treated mice spent shorter time in the target quadrant of the water pool in comparison to the untreated animals [28]. Contrary to this finding, our study on rats showed that treatment with AgNPs(BSA), AgNPs(PEG), and Ag^+^ had no effect on the long-term memory of animals in comparison to the Ctrl rats. The results suggest that AgNPs coated with BSA or PEG can modify the functional integrity of the hippocampus, which is responsible for relational binding associated with the formation of long-term memory [29]. Skalska et al. revealed that exposure to AgNPs may induce synaptic degeneration, as indicated by the observed ultrastructural changes such as blurred synapse structure and disorganized synaptic membrane, which in turn led to the release of synaptic vesicles into neutrophils [13].

In our study, we observed that application of BSA- and PEG-coated AgNPs resulted in the impairment of short-term memory, which is critical for assessing higher-level cognitive functions. Short-term memory reflects the mental ability to temporarily hold a limited amount of information that is no longer present in the sensory environment [30]. A study evaluated short-term memory by performing a novel object recognition test and reported that the memory was intact in mice intranasally treated with uncoated AgNPs [31]. It was shown that citrate buffer- or silver citrate-stabilized AgNPs intragastrically administered at a dose of 0.2 mg/kg b.w. for 14 days had no effect on object recognition memory in rats. Analysis of the effect of Ag^+^ on fear response using the plus maze test indicated that Ag^+^ induced a weak depressive effect and hyperalgesia, as observed by a longer time spent by mice in the open arms of the plus maze test in comparison to untreated mice and mice treated with citrate-coated AgNPs [31]. In agreement with our study, Dąbrowska-Bouta et al. noticed that neither Ag^+^ nor AgNPs with different types of coating had an influence on the locomotor activity. The memory retrieval test also did not reveal any differences in the locomotor activity between the groups [31].

Neurobehavioral disturbances may result from the direct and indirect action of AgNPs. These nanoparticles can accumulate in the gastrointestinal tract and then translocate to the brain through the bloodstream. Our previous studies have confirmed that AgNPs administered by intravenous and oral routes accumulated as silver in the brain [8,9]. When administered orally, AgNPs can either enter the brain and bloodstream and penetrate the BBB, or are transported to the brain via vagus nerve through retrograde axonal transmission [18,32]. Additionally, orally administered AgNPs or Ag^+^ released from nanoparticle surfaces can destroy the integrity of the intestinal wall and increase its permeability, thereby facilitating bacterial and food antigens to penetrate into the deeper layers of the intestine through the intestinal barrier. The resulting intestinal barrier damage can potentially induce an inflammatory response in the colon wall as well as cause changes in the gut microbiota [22]. As gut–brain axis plays an important role, AgNPs accumulation in the gastrointestinal tract and their effect on immune and microbiome components of the gut–brain axis may lead to neurobehavioral toxicity, as observed in the present study.

In the present study, we also evaluated the neurobehavioral effect of AgNPs with different types of coating by assessing the deferred changes in neurotransmitter levels in the rat hippocampus. Neurotransmitters involve in all brain functions by binding to specific receptors in defined brain structures. Therefore, alterations in the level of these substances may affect cognitive functions, memory, and locomotion. The hippocampus of the brain is fundamental for memory and cognitive functioning, and appears to be particularly susceptible to the adverse effects of AgNPs [9,13,33]. Our study revealed that, despite the results of behavioral test, there were no significant difference in the concentration of glutamic acid between the experimental groups, whereas its increase, according to the literature, is symptomatic for the memory acquisition [34]. This is likely due to the fact that the measurements of neurotransmitters levels in our experimental set up do not cover the memory acquisition period during the AAPA test. In our experiment, the neurotransmitters were assessed in hippocampi isolated at the end of the study (after the retrieval test and a few days after the end of the place avoidance training), whereas effective learning occurred between the first and third day of AAPA training. In contrast, the present study revealed that the impairment of higher brain functions which was observed after the administration of AgNPs or Ag^+^ could be due to long-term modifications of the levels of dopamine, serotonin, and acetylcholine. The concentration of serotonin in the AgNPs(Cit), AgNPs(BSA), and AgNPs(PEG)-receiving groups was lower than in the Ag^+^-receiving and the control groups. In addition, in AgNPs(BSA)-receiving rats, a low level of serotonin was associated with an overproduction of dopamine. Dopamine and serotonin system dysfunction in humans is linked to symptoms of depression, such as low motivation, emotions, and overall mood [35]. In animals, the depressive symptoms are described as anhedonia and anxiety-like behavior [36]. The AgNPs(BSA)-receiving rats presented also a skill learning impairment. Though the behavioral parameters measured in the AAPA task do not measure depression or anxiety-like symptoms, it might be speculated that that impairment of skill learning of AgNPs(BSA)-receiving rats could result from the anxiety.

In our study, we observed increased concentration of dopamine in the animals exposed to AgNPs(BSA) and Ag^+^, but not AgNPs(Cit) or AgNPs(PEG). Hadrup et al. also observed an increased brain dopamine concentration in whole-brain homogenate after 28 days of oral administration of Ag in ionic form and PVP-stabilized AgNPs [12]. Furthermore, Liu et al. indicated the effects of AgNPs on the brain dopaminergic functions [33], showing that exposure to AgNPs caused a significant increase in the expression of genes involved in dopaminergic transmission (SPP1, CACNA1S, and TACR3), which resulted in the dysregulation of dopaminergic transmission, increased intracellular calcium levels, and death of hippocampal CA1 neurons. The decrease in intracellular calcium levels results from the stimulation of dopamine receptors in the physiological state. The release of osteopontin (encoded by SPP1) during neuroinjury is associated with the onset of intracellular calcium precipitation in the brain structures, which occurs in degenerating neurons. Additionally, upregulation of TACR3, a gene that encodes neurokinin receptor-3 receptor, is related to intracellular calcium influx and cell death initiation, which altogether suggests that apoptosis is the principal mechanism of AgNPs-induced neurotoxicity [37].

Nevertheless, in our study, administration of AgNPs coated with citrate or PEG did not affect the level of dopamine. Dopamine-mediated neurotransmission in CNS supports motivation, spatial memory, and cognitive processes [38,39,40] and controls noncognitive processes, including locomotion [41]. In our study, the treatment had no effect on locomotor activity (data not presented). However, the same AAPAT showed that everyday blockade or stimulation of D2 dopamine receptors was dependent on the dosage and affected locomotor activity, but not cognitive performance. Administration of a D2 receptor agonist dose-dependently increased the locomotor activity, but had no effect on the performance in memory task [41]. Moreover, in the AAPAT, the cognitive performance was impaired by the antagonist (SCH23390) of D1 receptors, in contrast to the agonist (A77636) which improved the performance, while changes in locomotion were transient and likely had no effect on the performance [42]. Additionally, it was reported that AgNPs can reduce the level of inhibitory monoamines, such as gamma-aminobutyric acid and glycine, as well as modulate the functions of excitatory glutamic acid. Moreover, AgNPs can alter the expression of gene encoding glutamatergic N-methyl-D-aspartate receptor, which is a calcium channel in neurons, and the genes encoding monoamine oxidase A and B enzymes, which regulate the brain neurotransmitter levels [43]. This clearly suggests that the concentration of neurotransmitters in the brain is influenced not only by the dose, route, and duration of nanoparticles exposure [44] but also by the type of coating material applied on the nanoparticles.

Another goal of the present study was to identify the mechanisms underlying the long- and short-term memory impairment observed in our previous study. For this purpose, rats were exposed at a low dose to AgNPs coated with different materials. As the release of Ag^+^ from the nanoparticle surface is proposed as one of the main mechanisms of AgNPs toxicity, one group of rats were administered with Ag^+^ and the effect of the nanoparticles was compared with that of Ag^+^. In our previous study, we found that oral exposure to BSA-coated AgNPs at a low dose resulted in a significantly higher accumulation of silver in the hippocampus. Nevertheless, the nanoSIMS analysis revealed a weak silver signal in the hippocampus of the AgNPs-treated animals, which was assumed as related to the presence of silver in ionic form rather than in nanoparticulate form [9]. Several other studies have also reported that the neurotoxic effect of AgNPs was partially associated with Ag^+^, as these ions can disturb the mitochondrial respiratory chain affecting ATP production and resulting in its insufficiency, oxidative stress, and inflammation induction [45,46]. In our present study, we observed a slight systemic inflammation, including changes in the levels of proinflammatory cytokines and chemokines, after 28 days of oral exposure to AgNPs. The results thus demonstrated that exposure of animals to AgNPs coated with different material caused significant alterations in pro- and anti-inflammatory cytokines in plasma, which indicates the activation of inflammatory response. Oral administration PEG-coated AgNPs in rats resulted in a higher concentration of pro-inflammatory cytokines (IL-1β, IL-5, IL-6, IL-12(p70), TNF-α, GM-CSF, and G-CSF) as well as anti-inflammatory cytokines (IL-10). These changes in cytokines profile highlighted that PEG-coated AgNPs strongly stimulated immune response in comparison to the Ctrl group or animals treated with AgNPs coated with other coating materials. The higher plasma concentration of certain interleukins most likely indicates that immune system cells (such as macrophages, dendritic cells, or lymphocytes B) are stimulated upon exposure to PEG-coated AgNPs. Studies of other authors showed a lower release of TNF-α and IL-12, and a higher release of IL-10 and TGF-β from peripheral blood mononuclear cells upon exposure to PEG-coated AgNPs, indicating that this type of nanomaterial exhibits lower toxicity compared to bare AgNPs [47]. It has also been pointed out that peripheral inflammation can disrupt the integrity of the BBB [48] and, therefore, nanoparticle-induced increase in peripheral pro-inflammatory cytokines can affect the functioning of CNS and contribute to memory impairment [49]. Based on the statistical analysis of cytokine profiles of animals from different experimental groups, it can be assumed that the exposure to AgNPs coated with BSA moderately overstimulated the secretion of pro-inflammatory cytokines as compared to AgNPs coated with citrate and Ag+. Additionally, in the present study, ANOVA with multiple measurements revealed that the body weight changes in rats were significantly dependent on the interaction between time and type of exposure. In the present study, we did not intend to induce acute toxicity, which obviously would be associated with impaired brain function. The blood analysis revealed that all examined parameters were within physiological range [16] thus it is plausible to assume that administered dose had no systemic toxicity. Behavioral disturbances were observed along with changes in the hippocampal concentrations of neurotransmitters and slight systemic changes, which indicates the pro-inflammatory nature of the orally administered AgNPs. The obtained results indicate that the action of Ag^+^ ions and AgNPs differs, as was also confirmed by the results of Fisher’s LDA. The group administered with Ag^+^ was separated from both Ctrl group and nanosilver groups, suggesting the different mechanism of Ag^+^ action. Moreover, our results clearly showed that one of the factors modulating the neurotoxicity of AgNPs is the surface functionalization. A possible reason for the different behaviors of AgNPs coated with different types of materials can be the processes that the nanoparticles undergo in the gastrointestinal tract, which also include Ag^+^ release (probably in different amounts depending on the coating material used). Studies have described the impact of gastrointestinal fluids on the properties and fate of AgNPs. The characteristics of nanoparticles, including the coating material used for their stabilization, determine the transformation and bioavailability of the ingested particles [50,51,52]. The results of a study which analyzed the exposure of citrate-coated AgNPs to artificial human stomach fluid (pH 1.5) indicated that AgNPs significantly aggregate and release ionic silver, which reacts with the particle aggregates as silver chloride [53]. This agglomeration in the gastric fluid was also detected in the case of AgNPs with protein corona formed from BSA used as a food matrix component [52]. In this light, PEG is described as a biologically inert agent that decreases the affinity of nanoparticles for proteins and PEG-coated AgNPs are referred to as the most stable nanoparticles [23]. Taken together, it is clear that the fate of AgNPs can vary based on the nanoparticle properties, among which coating plays a key role.

## 4. Materials and Methods

### 4.1. Silver Nanoparticles Preparation and Characterization

AgNPs with a of nominal diameter of 20 ± 5 nm were acquired from PlasmaChem (Berlin, Germany). Nanoparticles preparation was carried out as described previously [54]. Briefly, a stock solution of nanoparticles was prepared by dispersing 2 mg of AgNPs in 800 μL of distilled water. Then, the AgNPs solution was sonicated on ice for 10 min using a probe sonicator (Branson, Danbury, CT, USA) with total ultrasound energy of 420 J·m^–3^. Immediately after sonication, 100 μL of 10× phosphate-buffered saline and 100 μL of 15% bovine serum albumin (BSA) were added. Sodium citrate-coated AgNPs with a diameter of 20 nm were purchased from NanoComposix (San Diego, CA, USA). Their hydrodynamic diameter was 25 nm and zeta potential was −43 mV as per the manufacturer’s data. Poly(ethylene glycol) methyl ether thiol (PEG; 5000 Da) was obtained from Sigma-Aldrich (St. Louis, MO, USA). PEG-coated AgNPs were fabricated by mixing 1 mg of AgNPs with 100 µL of SH-PEG aqueous solution (1 mg of PEG dissolved in 100 µL of water), and the resulting mixture was stirred for 2 h at room temperature. The hydrodynamic diameter and zeta potential (ζ) were measured by dynamic light scattering at 25 °C with a scattering angle of 173° using the Zetasizer Nano ZS system (Malvern, Malvern Hills, UK). Stock solutions of nanoparticles were diluted in water and measured in triplicate with 14 sub-runs. The suspensions had a pH value of 7.4. Zeta potentials were calculated using the Smoluchowski limit for the Henry equation, by applying a setting calculated for practical use (f(ka) = 1.5).

The characterization of AgNPs suspensions with different coatings studied in the in vivo experiment included the analysis of zeta potential, hydrodynamic size (Zetasizer Nano ZS; Malvern, Malvern Hills, UK), and aggregation state, as well as scanning electron microscopy (DSM 942; Carl Zeiss, Göttingen, Germany) and transmission electron microscopy analyses (JOEL 1200 EX II; JOEL, Tokyo, Japan) (Table 3).

### 4.2. Animals and Experimental Design

Ten-week-old male Wistar rats (outbred Cmdb:Wi) (*n* = 39) were obtained from Medical University of Bialystok, Center for Experimental Medicine (Polish Breeder’s register No. 003, GLP Certificate 16/2016/DPL). The rats had an initial body weight of 270 ± 9 g. The animals were placed in polyurethane cages in an animal house under standard conditions (lights on from 7:00 a.m. to 7:00 p.m., temperature 22 ± 1 °C, relative humidity 50 ± 5%, air exchange 15/h) and had access to feed (Labofeed B maintenance diet for laboratory rats, carbohydrates 67%, fat 8%, and protein 25% of metabolic energy according to AN93 recommendation; Kcynia, Poland) and water ad libitum. The animals were individually marked and grouped in four per cage throughout the experiment. All the study procedures were approved by the I Warsaw Local Ethics Committee for Animal Experimentation (Resolution No. 788/2015 of 25.05.2015) and performed in accordance with the guidelines of the European Communities Council Directive (Directive 2010/63/EU of 22 September 2010), Polish law, and 3R rule (Replacement, Reduction and Refinement).

**Figure 6:**
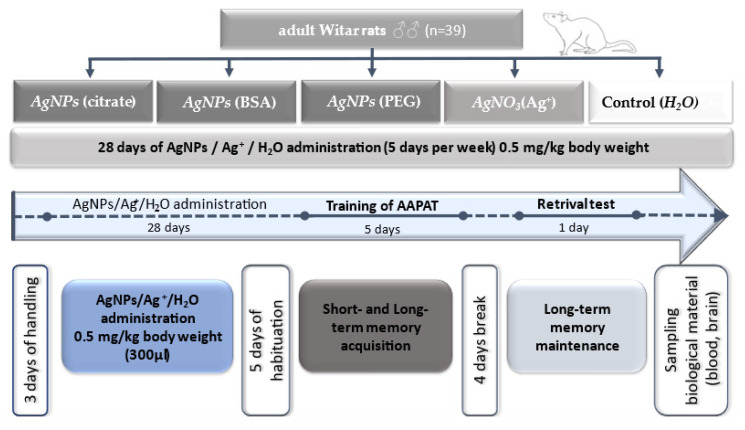
Experimental design and procedure scheme. Handling of rats was started 3 days before the administration of AgNPs/Ag^+^/H_2_O. The rats were habituated for behavioral training during the last 5 days of AgNPs administration.

After 10 days of acclimatization to the conditions of the animal house, the rats were randomly divided into five groups. The assessments were carried out in two turns, with four rats per turn per group. The animals were administered orally by gavage with (1) BSA-coated AgNPs (AgNPs(BSA)) (*n* = 8); (2) citrate-coated AgNPs (AgNPs(Cit)) (*n* = 8); (3) PEG-coated AgNPs (AgNPs(PEG)) (*n* = 8); or (4) AgNO_3_ as a source of Ag^+^ (*n* = 8). Another group of rats were used as control (Ctrl group) (*n* = 7). All groups were administered with 0.5 mg/kg b.w. of silver in 0.2 mL volume, whereas the Ctrl rats received 0.2 mL of water. AgNPs administration was performed once a day from Monday to Friday for 4 weeks. The experiment involved six stages as follows: (1) handling period, to allow the animals to accustom to the housing conditions and the experimenter (3 days); (2) treatment period, during which the animals were orally exposed to AgNPs or Ag^+^ (28 days); (3) habituation period, to allow the animals to accustom to the training procedure (during 5 days of the last week of AgNPs treatment period, the animals were habituated to the behavioral test by placing them on a stable arena without shocks for 10 min each time; (4) acquisition of short- and long-term spatial allothetic memory in the active allothetic place avoidance task (AAPAT) (one 20-min session per day for 5 days); (5) 4-day break; and (6) retrieval test to assess long-term memory (1 day). One week after the test, the rats were bled from the heart while under deep isoflurane ((Baxter Healthcare, Warsaw, Poland) anesthesia. The experimental design and timeline are presented in Figure 6.

### 4.3. Blood Collection, Hematological Analysis of Whole Blood, and Cytokine Profile in Plasma

The peripheral blood was collected in ethylene diamine tetra-acetic acid-coated test tubes from the heart of rats while under deep isoflurane anesthesia. The collected whole blood was centrifuged at 2200× *g* for 15 min at 4 °C, and the resulting plasma samples were stored at −20 °C until further analysis. Hematological analysis was performed in the whole blood using an Abacus Junior Vet analyzer (BioMaxima, Lublin, Poland). The analysis included the assessment of the following parameters of red blood cells (RBC): total number of RBC, mean corpuscular volume (MCV), red cell distribution width (RDWc), mean corpuscular hemoglobin concentration (MCHC; mean concentration of hemoglobin in erythrocytes), mean corpuscular hemoglobin (MCH; mean content of hemoglobin in a single erythrocyte) in the cells, and concentration of hemoglobin (HGB). In addition, immune cell parameters, such as the total number of white blood cells (WBC), monocytes and eosinophils (MID), lymphocytes (LYM), granulocytes (GRA), platelets (PLT), and hematocrit (HCT) were measured. The plasma levels of cytokines were determined using the Bio-Plex Pro^TM^ Rat Cytokine 24-Plex Assay (Bio-Rad, Hercules, CA, USA) in the Bio-Plex 200 System (Bio-Rad, Hercules, CA, USA). The final data were analyzed in the Bio-Plex 3D Suspension Array System (Bio-Rad). The assay allows estimating the plasma concentration of granulocyte stimulating factor (G-CSF), granulocyte macrophage colony stimulating factor (GM-CSF), growth-related oncogene (GRO/KC), interferon γ (IFN-γ), interleukin (IL) 1α, IL-1β, IL-2, IL-4, IL-5, IL-6, IL-7, IL-10, IL-12(p70), IL-13, IL-17A, and IL-18, monocyte colony-stimulating factor (M-CSF), monocyte chemoattractant protein 1 (MCP-1), macrophage inflammatory protein 1α (MIP-1α) and 3α (MIP-3α), tumor necrosis factor alpha (TNF-α), vascular endothelial growth factor (VEGF), and regulated on activation, normal T-cell expressed and secreted (RANTES).

### 4.4. Apparatus and Behavioral Procedure

Spatial memory of the rats was assessed by performing the AAPAT, as previously described [55]. Briefly, the testing system consisted of a rotating 80 cm aluminum arena flanked by a 1 cm peripheral metal rim. The arena was placed 80 cm above the floor at the center of a room containing many visual cues.

A virtual shock sector (60°) was set in a stable position to the distal room cues (Room+), whereas the proximal cues from the rotating arena were misleading (Arena−). The condition for generating conflict between the room and arena frames has been described in detail elsewhere [55]. An infrared light-emitting diode (LED) was attached to a latex harness on the back of each rat. The position of LED in the reference frame of the room was tracked every 20 ms by a computer system using an infrared-sensitive TV camera. A second infrared LED was fixed to the periphery of the arena to calculate the rat’s position in the reference frame of the arena. Thus, a to-be-avoided sector was defined in the reference frame of both room and arena. Before the experiment, the rats were implanted with a 25-gauge (0.50 mm) hypodermic needle, puncturing the skin fold on the back. The sharp end of the needle was then cut off, and a small loop was formed with tweezers. This loop was connected by a mini-alligator clip attached with a cable to a shock box used for delivering electric shocks. A computer system connected with an infrared TV camera was used to monitor the position of the rat every 20 ms. Whenever the rat entered the to-be-avoided sector, the computer system triggered a mild constant current (50 Hz, 0.5 s) foot-shock that was delivered across the low- and high-impendence electrodes. The low-impendence (~100 Ω) shock electrode was clipped to the needle loop on the rat’s back, while high-impendence (~100 kΩ) shock was produced when the rat’s feet contacted the ground in the arena surface. For each rat, the shock amplitude (0.2–0.5 mA) was adjusted so that its response to the shock was moderate and did not induce freezing or attempts to escape from the arena. If the rat stayed inside the shock sector, the shock was repeated at 1.5 s intervals until it escaped from the sector. The arena was located in a separate room to maintain constant experimental conditions, and the experiment was monitored from an adjacent room. Data collection and analysis were performed using commercial software (Biosignal Group, Acton, MA, USA) [55].

In the AAPAT, the shock sector was located in a fixed position relative to the relevant room distal cues, whereas the proximal cues (e.g., urine or defecation) from the arena were misleading (Room+Arena−). Thus, this task required the rats to use the reference frame of the room and ignore information from the arena frame. Four days following the acquisition training, the retrieval test on long-term memory recollection was conducted under the same conditions as memory acquisition, but did not involve shocks. Spatial memory acquisition was assessed on each of the 5 days of place avoidance training, by measuring the number of entrances into the to-be-avoided sector (ENTR) and the number of shocks received by the rat in a quarter of the arena with shock sector (SH). Moreover, on each day of the training, short-term memory functioning was evaluated by measuring the maximum time avoided (Tmax). Long-term memory was assessed by measuring the time to the first entrance to the to-be-avoided or shock sector (T1). Learning skills were assessed by calculating the ratio of the number of shocks per entrances (SHs/ENTRs). Performance of this task requires the formation of spatial memory of a to-be-avoided sector which demands the rats to segregate useful distal information from the room and misleading information from the arena and self-motion. The noncognitive memory, presented as spontaneous activity, was also examined during the assessment. Locomotion was measured as the locomotor activity of rats which is expressed by the total path length.

### 4.5. Neurotransmitters Content in the Hippocampus

The levels of neurotransmitters (acetylcholine, dopamine, glutamic acid, and serotonin) in the hippocampus were measured by quadrupole time-of-flight tandem mass spectrometry (SCIEX TripleTOF 5600+ DuoSpray), and identification was performed based on commercially available standards (Sigma-Aldrich, St. Louis, MO, USA). Briefly, rat hippocampus was homogenized with 800 µL of an acetonitrile–methanol mixture (1:1) and then vortexed (2000 rotations for 15 min) and centrifuged for 15 min at 13,000 rpm. The resulting supernatant was transferred to glass autosampler vials and placed in an autosampler at 4 °C. For chromatographic separation, a Hypersil chromatographic column (BDS C18, 150 × 4.6 mm, size 5 mm; Phenomenex, Torrance, CA, USA) along with a Hypersil C18 guard column (10 × 2.1 mm, size 5 μm) was used. The mobile phase was made of methanol–formic acid mixture (99:1, *v*/*v*; solvent A) and water–formic acid mixture (99:1, *v*/*v*, solvent B). The flow rate was set constant at 500 µL · min^−1^. The gradient elution of mobile phase was started at 100% A and proceeded as follows: 1.1–40 min linear gradient to 100% B, 40.1–55 min 100% B, and 55.1–60 min linear gradient to 100% A. The total runtime was 60 min. The optimized detection conditions for mass spectrometry (MS) were as follows: curtain gas (N2)—25 psi, nebulizer gas (N2)—20 psi, heater gas (N2)—50 psi, ion source voltage floating—5500 V, and source temperature—500 °C. Samples with a heated electrospray ionization (ESI) probe were measured in positive ionization mode (H-ESI+). Analysis of every third sample using the Calibrant Delivery System (SCIEX) MS system was auto-calibrated using original calibrators (SCIEX). Quantitative analysis and method validation were performed using the original SCIEX software (Analyst, PeakView, MasterView).

### 4.6. Statistical Analysis

Experimental data were analyzed using Statistica software v. 13.3 (StatSoft, Tulsa, OK, USA) for analysis of variance (ANOVA) and R statistical software v. 3.3.3 (www.rproject.org/ accessed on 6 October 2021) (R: The R Project for Statistical Computing) for Fisher’s Linear Discriminant Analysis (LDA). The results collected from behavioral tests and data on body mass acquisition were analyzed by a two-way ANOVA (groups vs. days with repeated measures on days) followed by a Tukey post hoc test. The measured hematological parameters and plasma cytokine concentrations were analyzed with a one-way ANOVA, with groups as a factor, followed by a Tukey post hoc test. Significance was set at a level of *p* < 0.05. The residual normality of the data was verified by Shapiro–Wilk’s test. Brown–Forsythe’s test was performed to determine homoscedasticity. The parameters which were non normally distributed or revealed heteroscedasticity among experimental groups were log-transformed before statistical evaluation. Results are presented as mean ± standard error of the mean (SEM) of nontransformed values.

## 5. Conclusions

The present study showed that the effect of nanosilver on cognitive functions depends on the coating material used for stabilization. Application of BSA- and PEG-coated AgNPs, as well as AgNO_3_ as a source of Ag^+^, was found to result in the impairment of cognitive functions. The substantial impairment of long-term memory, together with the impairment of memory acquisition, observed in rats from the AgNPs(BSA) group confirmed that memory formation in this group was disturbed during the formation of memory traces and their consolidation. Moreover, these rats did not improve their learning skills. Behavioral disturbances co-occurred with changes in the hippocampal concentrations of neurotransmitters as well as slight systemic changes, which indicates the pro-inflammatory nature of the orally administered AgNPs. The results of the study suggest that the mechanism of action of silver administered as ions (AgNO_3_ solution) differs from that of Ag^+^ released from AgNPs. However, further studies including both in vivo and in vitro experiments are necessary to assess the exact mechanism underlying the neurotoxicity of AgNPs.

## Figures and Tables

**Table 1:** Hematological values (mean ± SEM).

	Ctrl	AgNPs(Cit)	AgNPs(BSA)	AgNPs(PEG)	Ag^+^	ANOVA
RBC [10^12^/L]	8.18 ± 0.09	8.01 ± 0.15	8.14 ± 0.12	8.00 ± 0.11	8.21 ± 0.12	*F*_4,34_ = 0.65, NS
MCV [fL]	53.86 ± 0.46 ^a^	54.14 ± 0.83 ^b^	54.71 ± 0.64	53.50 ± 0.82	50.71 ± 0.68 ^a,b^	*F*_4,31_ = 4.70, *p* = 0.004
RDWc [%]	16.86 ± 0.27	17.03 ± 0.14	16.47 ± 0.18 ^a^	16.34 ± 0.12 ^b^	17.36 ± 0.17 ^a,b^	*F*_4,32_ = 5.16, *p* = 0.003
MCHC [g/dL]	31.29 ± 0.23	31.20 ± 0.18	31.53 ± 0.15	32.16 ± 0.27	32.02 ± 0.41	*F*_4,28_ = 2.80, *p* = 0.047
MCH [pg]	16.77 ± 0.15 ^a^	17.19 ± 0.16 ^b^	17.36 ± 0.23 ^b^	17.33 ± 0.17 ^b^	16.39 ± 0.20 ^a,b^	*F*_4,32_ = 5.02, *p* = 0.003
HGB [g/dL]	13.56 ± 0.14	13.78 ± 0.17	14.11 ± 0.08 ^a^	14.00 ± 0.14	13.56 ± 0.14 ^a^	*F*_4,29_ = 3.65, *p* = 0.016
WBC [10^9^/L]	5.85 ± 0.34 ^a^	7.66 ± 0.62 ^a^	7.12 ± 0.34	7.07 ± 0.49	6.21 ± 0.36	*F*_4,31_ = 2.82, *p* = 0.042
MID [10^9^/L]	0.21 ± 0.05	0.20 ± 0.03	0.19 ± 0.03	0.14 ± 0.03	0.22 ± 0.05	*F*_4,31_ = 0.76, NS
LYM [10^9^/L]	4.58 ± 0.29	6.06 ± 0.50	5.34 ± 0.38	5.68 ± 0.43	4.85 ± 0.26	*F*_4,32_ = 2.44, NS
GRA [10^9^/L]	0.97 ± 0.09	1.35 ± 0.17	1.22 ± 0.13	1.15 ± 0.10	1.14 ± 0.12	*F*_4,34_ = 1.16, NS
PLT [10^9^/L]	660.71 ± 33.14	641.13 ± 26.68	592.71 ± 33.75	621.13 ± 31.31	661.38 ± 30.91	*F*_4,33_ = 0.84, NS
PCT [%]	0.52 ± 0.03	0.51 ± 0.03	0.48 ± 0.04	0.49 ± 0.03	0.50 ± 0.03	*F*_4,34_ = 0.20, NS
PDWc [%]	33.44 ± 0.42	33.11 ± 0.49	33.34 ± 0.32	33.43 ± 0.39	32.74 ± 0.36	*F*_4,33_ = 0.56, NS
HCT [%]	43.85 ± 0.38	43.11 ± 0.82	43.75 ± 0.52	42.78 ± 0.62	42.25 ± 0.51	*F*_4,34_ = 1.60, NS

^a, b^ Statistically significant difference from the silver-exposed group according to the Tukey post hoc test (*p* < 0.05). The same letters indicate statistically significant results.

**Table 2:** Blood plasma cytokine concentration (mean ± SEM).

Cytokine	Ctrl	AgNPs(Cit)	AgNPs(BSA)	AgNPs(PEG)	Ag^+^	ANOVA
IL-1α	68.34 ± 9.59	78.74 ± 12.96	71.51 ± 8.89	107.68 ± 12.82	76.67 ± 12.02	*F*_4,33_ = 1.61, NS
IL-1β	33.59 ± 5.53	40.87 ± 5.13	24.18 ± 2.58 ^a^	64.84 ± 10.11 ^a^	37.32 ± 5.25	*F*_4,25_ = 6.47, *p* = 0.001
IL-2	669.97 ± 96.0	967.03 ± 175.88	785.48 ± 105.32	1224.81 ± 151.15	832.07 ± 154.2	*F*_4,32_ = 1.89, NS
IL-4	42.79 ± 6.34	56.65 ± 8.86	48.28 ± 4.37	75.50 ± 9.12	45.44 ± 5.25	*F*_4,33_ = 2.51, NS
IL-5	214.48 ± 21.72 ^a^	278.74 ± 20.58	242.78 ± 14.64	310.49 ± 22.32 ^a^	246.91 ± 9.25	*F*_4,28_ = 3.75, *p* = 0.01
IL-6	121.68 ± 18.17 ^a^	268.43 ± 12.56 ^a,b^	195.22 ± 28.92	338.21 ± 48.62 ^a,c^	247.79 ± 63.44	*F*_4,22_ = 5.60, *p* = 0.003
IL-7	73.89 ± 31.49	48.51 ± 5.56	34.66 ± 2.96	63.58 ± 8.68	39.11 ± 2.54	*F*_4,30_ = 2.63, NS
IL-10	32.90 ± 4.09 ^a^	56.69 ± 8.97	41.60 ± 3.37	68.96 ± 8.01 ^a^	43.61 ± 4.78	*F*_4,30_ = 4.58, *p* = 0.005
IL-12(p70)	62.48 ± 11.72 ^a^	186.77 ± 28.07 ^a,b^	119.91 ± 21.62	182.89 ± 27.35 ^a,c^	152.46 ± 28.50 ^a,d^	*F*_4,26_ = 7.50, *p* = 0.000
IL-13	87.18 ± 22.47	80.91 ± 14.44	85.56 ± 29.26	120.09 ± 23.40	99.76 ± 26.94	*F*_4,25_ = 0.48, NS
IL-18	3277.5 ± 799.5	2689.0 ± 441.8	2230.0 ± 206.2	3630.1 ± 658.9	3635.1 ± 644.4	*F*_4,32_ = 0.91, NS
IFN-γ	124.39 ± 25.57 ^a^	232.13 ± 21.96 ^a,b^	170.42 ± 20.55	283.98 ± 40.6 ^a,c^	190.41 ± 40.26	*F*_4,31_ = 4.28, *p* = 0.007
TNF-α	377.29 ± 59.88 ^a^	536.16 ± 55.77	467.36 ± 58.10	681.46 ± 71.91 ^a, b^	409.04 ± 49.27 ^b^	*F*_4,30_ = 4.26, *p* = 0.008
GRO/KC	36.14 ± 6.02	30.88 ± 3.61	22.99 ± 1.83	36.88 ± 4.08	34.00 ± 4.75	*F*_4,31_ = 2.51, NS
GM-CSF	31.80 ± 7.35	33.82 ± 5.25	23.67 ± 2.99 ^a^	54.52 ± 9.66 ^a^	29.40 ± 3.21	*F*_4,27_ = 3.67, *p* = 0.016
G-CSF	3.71 ± 0.63	5.92 ± 0.42	4.83 ± 0.81	8.35 ± 1.09 ^a^	3.22 ± 0.42 ^a^	*F*_4,29_ = 3.82, *p* = 0.013
M-CSF	10.54 ± 1.34 ^a^	15.89 ± 1.87	9.61 ± 0.46 ^b,c^	23.93 ± 2.55 ^a,b^	14.03 ± 3.02 ^c^	*F*_4,19_ = 8.08, *p* = 0.001
MCP-1	390.18 ± 25.44	321.45 ± 26.85	296.23 ± 17.42	315.59 ± 25.02	371.18 ± 16.28	*F*_4,32_ = 2.90, *p* = 0.037
MIP-3α	19.15 ± 1.50 ^a^	19.66 ± 1.13 ^b^	19.39 ± 1.47 ^c^	20.97 ± 0.80	29.29 ± 3.43 ^a,b,c^	*F*_4,29_ = 4.63, *p* = 0.005
MIP-1α	1.05 ± 0.06	0.98 ± 0.05	0.89 ± 0.04	1.07 ± 0.04	0.99 ± 0.01	*F*_4,29_ = 2.58, NS
RANTES	98.71 ± 8.46	81.98 ± 8.59	91.91 ± 16.15	75.90 ± 6.44	110.28 ± 12.96	*F*_4,29_ = 1.47, NS

^a–d^ Statistically significant difference from the silver-exposed group according to the Tukey post hoc test (*p* < 0.05). The same letters indicate statistically significant results.

**Table 3:** Characterization of AgNPs after dispersion in water (modified based on Ref. [54]).

	BSA-Coated AgNPs	PEG-Coated AgNPs	Citrate-Coated AgNPs
Nominal size of Ag particles (nm)	20 ± 5	25 ± 5	25 ± 5
Dynamic light scattering (nm)	84.4 ± 3.7	58.3 ± 6.5	27.5 ± 5.6
Polydispersity index	0.295	0.144 ± 0.06	0.308 ± 0.05
Zeta potential (mV)	–33.6	–30.2	–32.5

Data are expressed as mean ± SD, *n* = 3.

## Data Availability

The data that support the findings of this study are available on request from the corresponding author (K.D.).
